# Comparative Analysis of WUSCHEL-Related Homeobox Genes Revealed Their Parent-of-Origin and Cell Type-Specific Expression Pattern During Early Embryogenesis in Tobacco

**DOI:** 10.3389/fpls.2018.00311

**Published:** 2018-03-08

**Authors:** Xuemei Zhou, Yingying Guo, Peng Zhao, Meng-xiang Sun

**Affiliations:** State Key Laboratory of Hybrid Rice, College of Life Sciences, Wuhan University, Wuhan, China

**Keywords:** WUSCHEL-related homeobox gene, early embryo, apical cell, basal cell, transcription repressive activity, tobacco

## Abstract

WUSCHEL-related homeobox (WOX) gene is a plant-specific clade of homeobox transcription factors. Increasing evidences reveal that WOXs play critical roles in early embryogenesis, which involves zygote development, initiation of zygote division, and apical or basal cell lineage establishment. However, how *WOXs* regulate these developmental events remains largely unknown, and even detailed expression pattern in gametes and early proembryos is not yet available. Here, 13 *WOX* family genes were identified in *Nicotiana tabacum* genome. Comparative analysis of 13 *WOX* family genes with their homologs in *Arabidopsis thaliana* reveals relatively conserved expression pattern of *WUS* and *WOX5* in shoot/root apical meristem. Whereas variations were also found, e.g., lacking homolog of *WOX8* (a marker for suspensor cell) in tobacco genome and the expression of *WOX2*/*WOX9* in both apical cell and basal cell. Transient transcriptional activity analysis revealed that WOXs in WUS clade have repressive activities for their target's transcription, whereas WOXs in ancient and intermediate clade have activation activities, giving a molecular basis for the phylogenetic classification of tobacco WOXs into three major clades. Expression pattern analysis revealed that some *WOXs* (e.g., *WOX 13a*) expressed in both male and female gametes and some *WOXs* (e.g., *WOX 11* and *WOX 13b*) displayed the characteristics of parent-of-origin genes. Interestingly, some *WOXs* (e.g., *WOX2* and *WOX9*), which are essential for early embryo patterning, were *de novo* transcribed in zygote, indicating relevant mechanism for embryo pattern formation is only established in zygote right after fertilization and not carried in by gametes. We also found that most *WOXs* displayed a stage-specific and cell type-specific expression pattern. Taken together, this work provides a detailed landscape of *WOXs* in tobacco during fertilization and early embryogenesis, which will facilitate the understanding of their specific roles in these critical developmental processes of embryogenesis.

## Introduction

Homeobox (HB) protein is a larger superfamily of eukaryotic transcription factors, which was first discovered in *Drosophila melanogaster*, and subsequently in many other eukaryotic organisms, ranging from sponges to vertebrates and mammals (Gehring et al., 1994). Homeobox proteins share a common homeodomain (HD) composed of 60 amino acids, which could recognize their binding sequences and regulate the expression of targeted genes in a precise spatial and temporal pattern, and exert various roles in different developmental processes, such as controlling of axial morphology and specifying segmental identity (Gehring et al., 1994; Pearson et al., 2005). Different types of HB genes have also been identified in plants, which exhibit diverse roles in plant developmental processes (Vollbrecht et al., 1991; Jain et al., 2008; Mukherjee et al., 2009). Among them, WUSCHEL-related homeobox (*WOX*) genes are distinguished as a plant-specific clade of HB transcription factors by phylogenetic relatedness of its HD from other HB transcription factors (van der Graaff et al., 2009).

*WOX* family genes were found to be ubiquitous present in the genomes of different plants, ranging from green algae to angiosperms (van der Graaff et al., 2009). However, the number of *WOXs* vary in plant genomes. *WOX* members expanded as the evolution of plants. There is only one *WOX* in the genome of green algae, whereas over 10 *WOXs* have been identified in the genome of angiosperms (Mukherjee et al., 2009). Phylogenetic analysis of *WOX* family genes from different species revealed that *WOXs* could be divided into three major clades including WUS clade, intermediate clade and ancient clade. Phylogenetic differences of *WOX* family genes between monocots and eudicots or between angiosperms and gymnosperms have also been revealed, respectively (van der Graaff et al., 2009).

Researches about *WOX* family genes in *Arabidopsis thaliana* revealed multiple roles of *WOX* family genes in different developmental processes, such as embryo patterning (Breuninger et al., 2008), stem-cell maintenance (Schoof et al., 2000; Leibfried et al., 2005; Forzani et al., 2014), floral transition (Lenhard et al., 2001), and so on. Fifteen *WOX* family genes have been identified in *A. thaliana*. Expression pattern analysis of them revealed that different members of *WOX* family display distinct expression pattern and comprise earliest cell fate markers in early embryogenesis (Haecker et al., 2004). *WOX2* and *WOX8* were found to be co-expressed in the zygote and confined to the apical cell and basal cell after zygote division, respectively. *WOX9* was firstly detected in the basal cell and subsequently shifted into the descendants of the apical cell (Haecker et al., 2004). *WOX2* was approved to act redundantly with *WOX1, WOX3*, and *WOX5* in embryo patterning. *WOX8* and *WOX9* were essential for development of apical and basal lineages of proembryo (Breuninger et al., 2008). After the establishment of the apical-basal pattern, another two *WOX* family genes, *WUS* and *WOX5*, could be detected in the shoot apical meristem (SAM) and root apical meristem (RAM), respectively (Schoof et al., 2000; Leibfried et al., 2005; Forzani et al., 2014).

Comparative analysis of *WOX* family genes between monocots and eudicots revealed that the expression pattern of several *WOX* family genes in SAM and RAM was partly similar, and the expression of these monocot orthologous genes appeared significantly delayed. Significant expression of *ZmWOX2A* and *ZmWOX9A/B* could only be detected in embryos at early transition stage. The expression of *WUS* and *WOX5* (*ZmWUS1* and *ZmWOX5*) in *Zea mays* were detected as early as embryo already consists of more than 100 cells (Zhao et al., 2017). Some other variations in *WOX* family genes between monocots and eudicots have also been found. The most striking divergence is that no *WOX8* has been identified in monocots to date. No similarities on the expression pattern of *WOX4* from *Z. mays* and *A. thaliana* were detected (Nardmann et al., 2007). All these results implied that the expression of *WOX* family genes has undergone modifications during plant evolution.

Given important roles of *WOX* family genes in early embryogenesis, the detailed expression pattern of *WOXs* is a prerequisite for elucidating their specific roles in embryo initiation and cell fate determination during early embryogenesis. Although considerable works have been done in *A. thaliana*, the detailed expression pattern of all members of *WOX* family in gametes, zygotes, and apical/basal cells is not yet investigated in any species. This expression pattern is necessary for evaluating the usability of these cell type-specific markers and elucidating the mechanism of embryo initiation and embryonic cell fate determination. Here, *WOX* family genes were firstly identified in *Nicotiana tabacum*, a model plant in eudicot clade, which displays a highly stereotyped, comparable and tractable cell division pattern during embryogenesis. Comparative analyses between *N. tabacum* and *A. thaliana* were carried out to gain insight into their conservation and differences. Especially, their specific expression in gametes and early proembryos was carefully investigated, providing a dynamic landscape of *WOXs* expression during fertilization and early embryogenesis.

## Materials and methods

### Plant materials

*N. tabacum* were grown in a 25°C greenhouse under 16/8 h light/dark cycle. *Nicotiana benthamiana* were grown in a 21°C greenhouse under 14/10 h light/dark cycle.

### Identification of WUS homeobox-containing (WOX) proteins

To identify *WOX* family genes in *N. tabacum*, the program tBlastn using WOX family protein sequences from *A. thaliana* were performed in the draft genomes, mRNA, and EST sequences of *N. tabacum*, respectively. DNA fragments and mRNA sequences related to *WOX* family genes were collected and assembled using the ContigExpress program with a minimum overlap of 100 bases and above 90% sequence identity in the overlap region. After assembly, redundant contigs related to *WOX* family genes were removed manually, and open reading frame (ORF) analysis of each contig was performed using OMEGA. Contigs with intact ORF were selected for further BLASTP analysis in National Center for Biotechnology Information. Contigs with HD domain were as *WOX* family candidates. Then, full-length cDNA of each candidate was obtained through reverse transcription-PCR (RT-PCR) with gene-specific primers at 3′ end and 5′ end, respectively.

### Protein sequence and phylogenetic analysis

To detect conserved domain of WOX family proteins, a multiple sequence alignment of protein sequences was conducted using the Clustal W program using the default multiple alignment parameters. Conserved motifs in WOX were analyzed on http://weblogo.berkeley.edu/logo.cgi. Signal peptide and nuclear localization signal predication were executed on SignalP server (Petersen et al., 2011) and cNLS Prediction, respectively. Three-dimensional structure of each WOX was predicated on SWISS-MODEL workspace (Arnold et al., 2006; Kiefer et al., 2009).

To construct a phylogenetic tree of WOXs from different plants, a genome-wide survey of *WOX* family genes in *Glycine max, Oryza sativa, Z. mays*, and *Sorghum bicolor* genomes was firstly executed, and sequences related to *WOX* family genes were collected. Then, a multiple sequence alignment of 84 WOX protein sequences from six different species was conducted using the Clustal X Ver. 1.81 program using the default multiple alignment parameters, and a phylogenetic tree was constructed with MEGA 5.1 using Neighbor-Joining Method.

### Embryo isolation and cDNA synthesis

Living sperm cell, egg cell, and embryo isolation were conducted according to the previous procedure (Zhao et al., 2014). To evaluate cell viability, embryos were stained with fluorescein diacetate (FDA). Isolated embryos were incubated in a solution containing 11% mannitol and 2 μg ml^−1^ FDA for 15 min at room temperature and washed twice with 11% mannitol before observation (Zhao et al., 2013). Intact mRNA was directly isolated from embryos using Dynabeads mRNA DIRECT^TM^ Micro Kit (Life Technologies, USA), and cDNA synthesis and amplification were performed with SMARTer™ Pico PCR cDNA Synthesis Kit (Clontech, USA).

### RNA isolation and RT-qPCR

Total RNA of leaf, root, stem, anther, pollen, pollen tube, petal, and sepal were extracted using TRI Reagent Solution (Life technologies, USA), and total RNA of seeds were extracted with RNAqueous^TM^ (Life technologies, USA). cDNA preparation from total RNA was conducted according to the previous procedure (Zhao et al., 2014). Quantitative real-time reverse transcription-PCR (RT-qPCR) was applied to analyze the expression pattern of *WOX* family genes. RT-qPCR was performed in a 10 μl mixture containing 5 μl 2×FastStart Essential DNA Green Master (Roche, Germany), 250 nM each primer, and cDNA templates Table S2. RT-qPCR was performed as the following procedure: activation of FastStart TaqDNA polymerase at 95°C for 10 min, and 45 cycles (95°C for 15 s, annealing at 60°C for 20 s; extension at 72°C for 30 s) on CFX-Connect Real-Time system (Bio-Rad, USA). Data processing was performed according to our previous protocol (Ma et al., 2011). Each data represent the mean ± standard error from three independent experiments.

### Subcellular localization of WOXs

To investigate the subcellular localization of each WOX, *35S:: YFP-NOS* vector was firstly constructed in pCAMBIA1300 to generate *pCAMBIA-35S::YFP-NOS*. Then, each *WOX* coding sequence (without stop codon) was cloned into the vector *pCAMBIA-35S::YFP-NOS* to generate *35S::WOX-YFP-NOS* expression vector. All vectors were transferred into *Agrobacterium tumefaciens* strain GV3101, and co-expressed with a nucleus marker H2B-CFP in *N. benthamiana* according to Sparkes (Sparkes et al., 2006). Transformed leaf epidermal cells were observed using a confocal microscope (Leica TCS SP8, Germany).

### Transient transcriptional activity analysis

The reporter vector *35S::GAL4-FLUC-NOS* and reference vector *35S::RLUC-NOS* was constructed in the pCAMBIA1300, respectively. To generate effector vectors, *35S::GAL4BD-NOS* was firstly constructed in pCAMBIA1300 to generate *pCAMBIA-35S::GAL4BD-NOS*. Then each *WOX* coding sequence (without stop codon) was cloned in-frame with GAL4BD into the vector *pCAMBIA-35S::GAL4BD-NOS* to generate *35S::GAL4BD-WOX-NOS* expression vector. All vectors were transferred into *Agrobacterium tumefaciens* strain GV3101 for transient expression. Agrobacterium solutions were then mixed at an OD_600_ of 0.1 for reporter and effector vectors, and an OD_600_ of 0.02 for the reference vector. A standard size of the Agrobacterium-infiltrated leaf was collected and ground in 250 μl of 1×Passive Lysis Buffer (Promega, USA) for protein extraction. Relative luciferase activity (FLuc/RLuc) of each effector was analyzed using Dual-Luciferase Reporter Assay (Promega, USA). Transcriptional activity analysis experiment was repeated four times. Each data represents the mean ± standard error from four independent experiments.

### Transcriptional activation analysis in yeast

For transcriptional activation assay, each *WOX* coding sequence (without stop codon) was cloned in-frame with GAL4 DNA binding domain in pGBKT7 to construct *pGBKT7-WOX*. pGBKT7 empty vector was used as a negative control. Different vectors were transformed into yeast strain AH109, respectively. These transformants were inoculated on the SD/Trp- and SD/Trp-/His-/Ade- medium. After incubated at 28°C for 6 days, the growth status of each transformant was checked. This experiment was repeated independently three times.

## Results

### Characterization of early embryogenesis in tobacco

**Figure 1:**
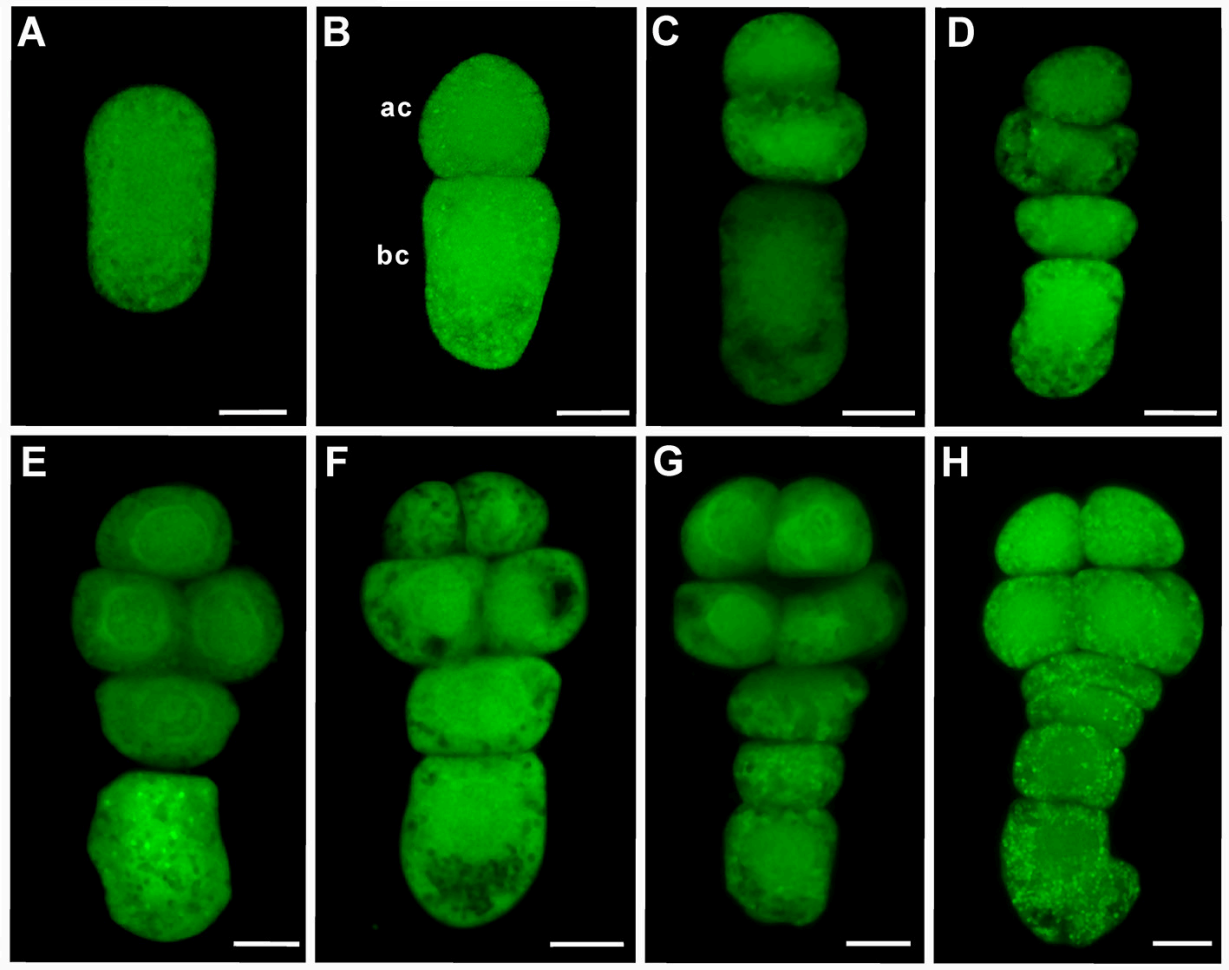
The process of early embryogenesis in tobacco. **(A)** Elongated zygote, **(B)** Two-celled proembryo, **(C)** Three-celled proembryo, **(D)** Four-celled proembryo, **(E)** Five-celled proembryo, **(F)** Six-celled proembryo, **(G)** Eight-celled embryo with a three-celled suspensor, **(H)** Eight-celled embryo with a four-celled suspensor. Embryos were stained with FDA. Ac: apical cell; Bc: basal cell. Bar = 10 μm.

To elucidate the dynamic expression of *WOX* family genes with a special concern about early events of embryogenesis, *N. tabacum*, a typical model plant for the study of embryogenesis (He et al., 2007; Zhao et al., 2013, 2016), was chosen for detailed analysis. Division pattern of the zygote and its daughter cells was carefully observed. First asymmetric zygote division leads to the formation of a small apical cell and a larger basal cell as that in *A. thaliana* (Figures 1A,B). The small apical cell will divide earlier than the larger basal cell, giving rise to the formation of a three-celled proembryo (Figure 1C). Later, the larger basal cell divide transversely, leading to the formation of a four-celled proembryo (Figure 1D). Subsequently, two daughter cells of the basal cell divide transversely, giving rise to the formation of a four-celled suspensor at eight-celled embryo stage (Figures 1E–H). These observations confirm that tobacco is an ideal model eudicot for the study of embryogenesis, which displays a highly stereotyped and predictable cell division pattern.

### Identification of *WOX* family genes in tobacco

To identify *WOX* family genes in tobacco, a tBLASTn search was carried out using *A. thaliana WOX* protein sequences in the draft genome sequences, mRNA sequences and EST sequences deposited in NCBI, respectively. Returned sequences related to *WOX* family genes were collected and assembled. After removing redundant sequences, 13 independent contigs were obtained. cDNA sequence of each contig was further confirmed by RT-PCR with gene-specific primes. ORF analysis revealed that each of them has a complete ORF of 555–1149 nucleotides (Table 1). BLASTP searches using their deduced protein sequences in NCBI returned several WOX family proteins from different species, implying that these genes are indeed members of *WOX* family genes. Thus, they were nominated according to the name of their sequence homology in *A. thaliana*. There are two striking features of *WOX* family genes in tobacco genome. The first is that several orthologs to *AtWOX6, AtWOX7, AtWOX8, AtWOX10, AtWOX12*, and *AtWOX14* have not been identified in tobacco genome. The second is that two *WOXs* (*NtWOX3* and *NtWOX13*) have multiple paralogs derived from chromosomal duplication. To investigate their genomic structures, a blastn search using full-length cDNA of each *WOX* was executed in the draft genome sequences. Genomic sequences related to each *WOX* were collected and compared with their cDNA sequences, respectively. The results revealed that each cDNA has its corresponding genomic sequence, which indicated that these *WOX* family genes really exist in tobacco genome. In addition, each *WOX* gene displays a common feature that the exons were separated by introns in the genome (Figure S1). However, the number of introns display substantial variations, varying from one to three. Compared to the conserved exon size (109–560 bp), the length of introns were more divergent, varying from 93 bp to 2 kb (Figure S1). It is also noteworthy that the highly conserved HD is usually located in the first or the second exon, and not separated by the insertion of introns.

**Table 1:** Detailed information for WOX family proteins in tobacco.

**Name**	**ORF (bp)**	**Predicated protein information**						
		**Position of HD domain**	**Position “LFP”**	**No. of amino acids**	**Mol. Wt (kDa)**	**isoelectric point**	**Signal peptide**	**Nuclear location signal position**
NtWOX1	1,149	76	333	383	43.4	7.2	–	136
NtWOX2	738	15	174	246	28.0	8.6	–	133
NtWOX3a	588	1	182	196	22.7	9.4	–	–
NtWOX3b	678	9	195	226	25.9	9.4	–	–
NtWOX3c	537	1	165	179	21.2	10.0	–	–
NtWOX3d	555	1	164	185	21.3	9.3	–	–
NtWOX4	705	92	225	235	26.8	9.2	–	192
NtWOX5	549	21	135	183	21.0	6.9	–	81
NtWOX9	1,143	47	–	381	42.1	7.3	–	–
NtWOX11	798	20	162	266	28.8	5.5	–	–
NtWOX13a	732	88	–	244	27.6	5.4	–	–
NtWOX13b	810	87	–	270	30.2	5.5	–	–
NtWUS	933	47	250	311	35.2	6.6	–	–

### Phylogenetic analysis of WOXs from different species

To examine the evolutionary relationship of the *WOX* family genes, the sequences related to *WOX* family genes from *G. max, O. sativa, Z. mays*, and *S. bicolor* were extracted firstly. Due to the lack of consensus nomenclature of *WOX* family genes, there are various names for *WOXs* in different publications and databases, leading to the confusion for distinguishing the some member of *WOX* family genes from that in other species. To make less confusing, we nominated each *WOX* gene derived from different species according to the name of its homology in *A. thaliana*, which was discovered earliest and explored most extensively (Table S1). Sequence alignment of HD protein sequences from 84 *WOXs* identified from *N. tabacum, A. thaliana, G. max, O. sativa, Z. mays*, and *S. bicolor* were performed as shown in Figure S2. Twelve residues in HD were completely conserved among all 84 WOXs, indicating a high degree of evolutionary conservation of WOXs. Then, phylogenetic tree based on HD sequences from *N. tabacum, A. thaliana, G. max, O. sativa, Z. mays*, and *S. bicolor* was constructed using neighbor-joining method. Generally, phylogenetic tree of WOX family proteins could be divided into three clades including WUS clade, intermediate clade and ancient clade (Figure S3). WUS clade is the largest clade composed of 49 WOXs including WUS and WOX1-7 derived from different species. The second subgroup is the intermediate clade composed of WOX8, WOX9, WOX11, and WOX12. The smallest subgroup is the ancient group which only contains three WOXs (WOX10, WOX13, and WOX14). Significant divergence exists in the WOX8/9 branch; no close *AtWOX8* relative exists in both monocots and eudicots in the phylogenetic tree, whereas all genomes contain *AtWOX9* orthologs. In addition, some other *WOXs* including (*WOX6, WOX7, WOX10*, and *WOX14*) were also lost in tobacco genome. Despite the loss of several individual *WOXs*, duplication of the tobacco genome is evident as that two tobacco *WOX* family genes (*NtWOX3* and *NtWOX13*) have multiple paralogs derived from chromosomal duplication within the genome, which has also been found in other plants such as *WOX2* and *WOX5* in maize (Figure S3 and Table S1). In addition, the distribution of *WOX*s is divergent. Nine *WOXs* fall into the WUS clade, and two *WOX*s fall into the intermediate (*NtWOX9* and *NtWOX11*) and ancient clade (*NtWOX13a* and *NtWOX13b*), respectively.

### Characterization of *WOX* family genes in tobacco

To characterize WOX family proteins in detail, the basic features and functional motifs of each WOX were investigated. The length of WOX protein sequences in tobacco is divergent, ranging from 183 amino acids for NtWOX3c to 383 amino acids for NtWOX1, with theoretical molecular weight ranging from 21.0 to 43.4 kDa (Table 1). To identify conserved motifs of WOXs, the protein sequences of 13 WOXs were aligned, and two conserved region motifs have been identified (Figures S4, S5). Generally, the overall structure of WOXs was highly conserved, with an HD located in the N-terminal region, and a WUS-box motif in the C terminus (Figure S5). Conserved HD is usually composed of 66 residues, which usually fold into a DNA-binding domain. There are 14 positions in HD that are occupied by the same amino acids in tobacco WOXs (Figure S4). The WUS-box motif is another hallmark for WOXs, which could be detected in the carboxy-terminal of most WOXs with three exceptions (NtWOX9, NtWOX13a, and NtWOX13b) (Figure S4). However, the positions of HD and WUS-box motif in the proteins are divergent. The HD domain of most WOX proteins, with three exceptions (NtWOX4, NtWOX13a, and NtWOX13b), is located in the N-terminal, whereas the HD domain of NtWOX4, NtWOX13a, and NtWOX13b are located in the middle of proteins. In contrast to the position of HD domain, the WUS-box motif of most WOXs except NtWOX11 was found in the same relative position in the carboxy-terminal (Figure S5). Another EAR-like motif (SLELSL), possibly involved in transcriptional repression (Ohta et al., 2001), could only be detected in the C-terminal region of WUS.

WOX is a specific subclade of HB transcription factor in plants, which is characterized by the presence of HD that could recognize and bind to distinct DNA sequences. In animals, three-dimensional structures of homeodomain-DNA complex and the mechanisms of HDs selecting their targeted DNA sequences have been comprehensively analyzed. Interactions between HD and their targeted DNA sequences are achieved by the “recognition” helix in DNA major groove and the N-terminal arm in the minor groove. However, DNA binding sequences of HD in WOXs and the mechanism for recognizing their targets in plants are still largely unknown. To predicate potential DNA targets of WOXs, Clustal X alignment of 72 typical HDs identified in *D. melanogaster* and 13 HDs from tobacco was performed (Figure S6), and three-dimensional structure of HDs was modeled according to the alignment mode as implemented in SWISS-MODEL (Figure S7). HDs in animals are usually composed of 60 amino acids that fold into a stable three-helix bundle preceded by a flexible N-terminal arm. Helices II and III are linked by a tight turn, and then give into a helix-tum-helix motif which recognizes their targeted DNA sequences. Homology modeling of HDs revealed that HD of WOXs display a conserved three-dimensional structure consisting three helixes connected by two loops (Figure S7). Alignment of HDs from the *D. melanogaster* and *N. tabacum* revealed that the HDs from *N. tabacum* are usually longer than that in *D. melanogaster*. Four additional amino acids were found in the second loop between Helices II and III (Figure S6), which could also be detected in WOXs from other plants (Figure S2). However, the role of the four-amino-acid loop in WOXs is still unknown.

### Expression profile of *WOXs* in tobacco

**Figure 2:**
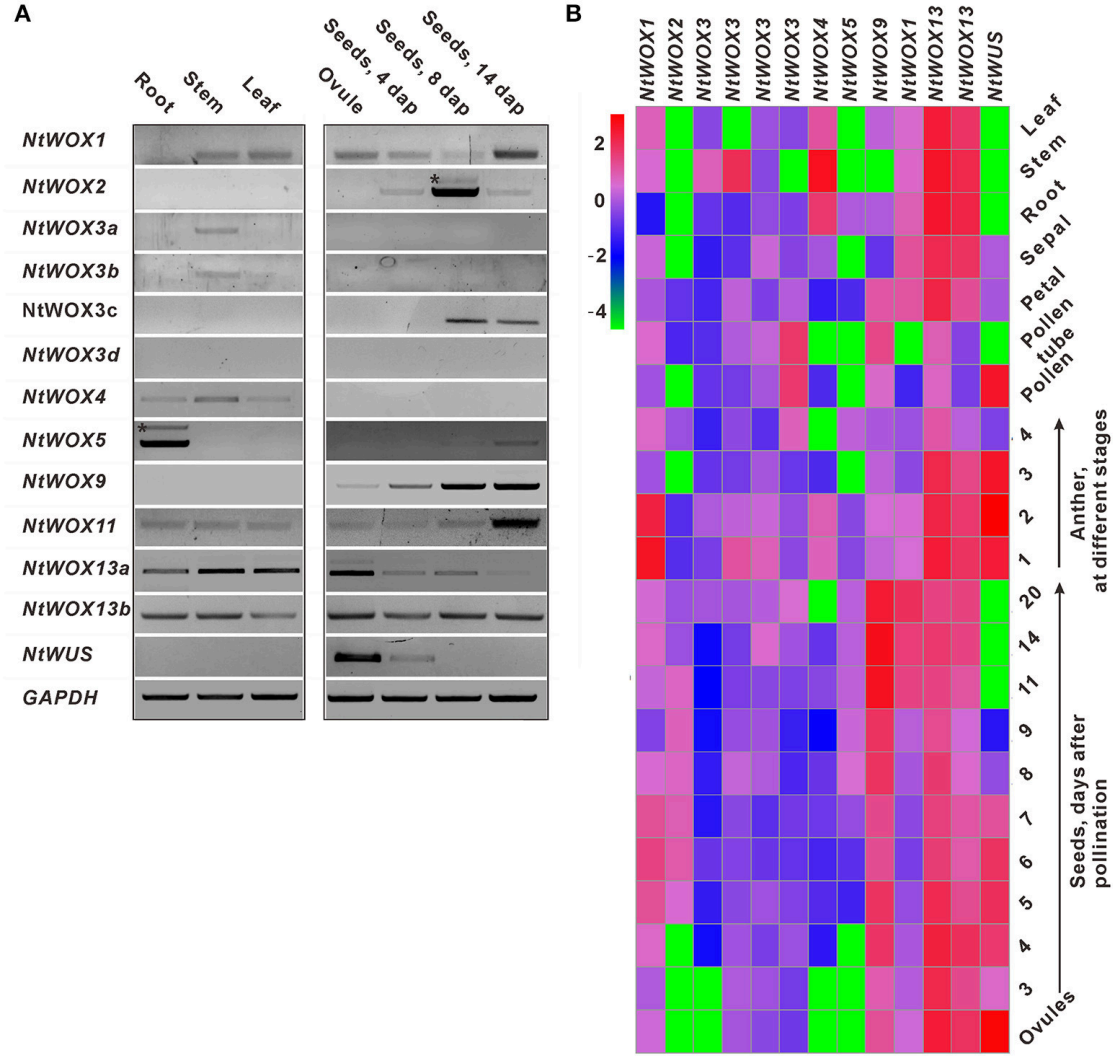
Expression pattern analysis of *WOXs* in tobacco. **(A)** RT-PCR analysis of the expression of *WOXs*. cDNA prepared from root, stem, leaf, ovule, and seeds at different stages were selected as templates for PCR. *GAPDH* was used as the control. ^*^ indicate no specific bands. **(B)** Expression profile of *WOX*s in tobacco, which was constructed based on the relative expression level of *WOXs* in different tissues. The expression level was normalized to the average expression level of GAPDH (AJ133422), polyubiquitin (GQ281244). Each data represents the mean of three biological repeats. A red box indicates a higher expression level, whereas a green box indicates the lower expression level. Anthers at stages 1–4 correspond to anthers containing microspore mother cells, tetrads, single-nucleated pollen, and bi-nucleated pollen, respectively. The scale bar represents the fold change (log10 value).

To gain insight into the expression profile of *WOXs*, RT-PCR was firstly carried out using cDNA template from leaf, stem, root, ovule, and seed. *GAPDH* was used as the internal reference for PCR. The transcripts of most *WOXs* with one exception (*WOX3d*) could be detected in one or more tissues tested (Figure 2A). Interestingly, four *WOXs* (*WOX2, WOX3c, WOX9*, and *WUS*) display a seed-specific expression pattern. To explore their expression pattern in detail, the relative expression level of each *WOX* in different tissues were detected through RT-qPCR. Heatmap analysis based on their relative expression levels was carried out, and an overview of the expression pattern of *WOXs* in tobacco is presented. Most *WOXs* display a tissue-specific expression pattern, such as *WOX2* which could only be detected in seeds. However, some other *WOXs* (such as *WOX11, WOX13a*, and *WOX13b*) display a relatively broad expression pattern, which could be detected in most tissues tested (Figure 2B).

### *WOXs* display a stage-specific expression pattern during the process of embryogenesis

**Figure 3:**
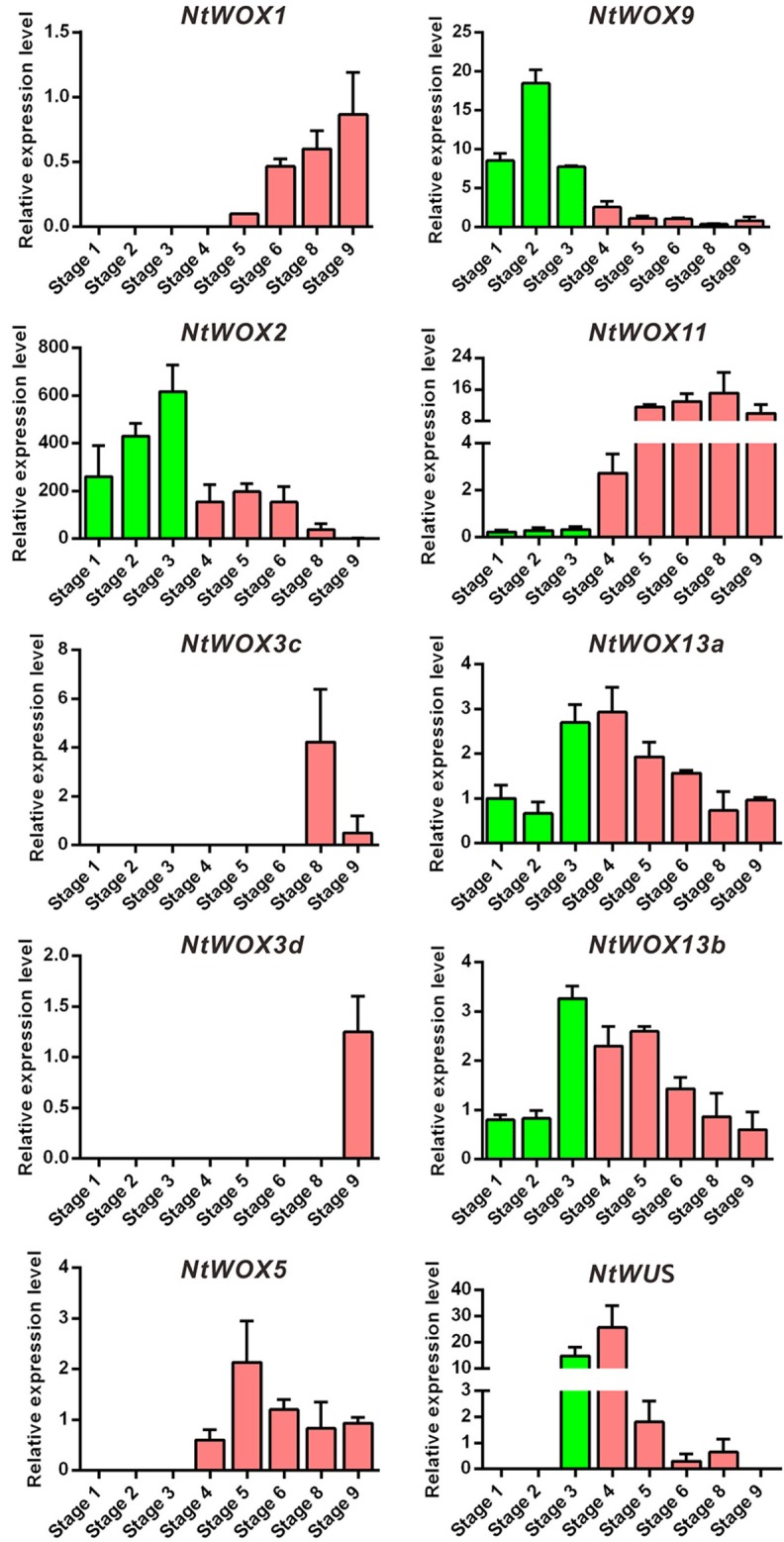
Stage-specific expression pattern of *WOXs* during the process of embryogenesis. The expression level of each *WOX* in embryo at stages 9 (except *WUS*) was set as 1. The expression level was normalized to the average expression level of GAPDH (AJ133422), polyubiquitin (GQ281244), and elongation factor 1α (AF120093). Error bars represent mean ± standard error from three independent experiments. Nine successive stages of embryogenesis were classified in tobacco as described previously (Zhao et al., 2013).

Expression pattern analysis of *WOXs* in *A. thaliana* revealed an outstanding expression characteristics of *WOXs* during embryo development: cell type-specific and stage-specific expression programs. In *A. thaliana*, it was reported that *WOX9* expression was initiated in the basal cell and subsequently shifted into embryo proper. *WOX5* which marks the quiescent center was initiated in the hypophysis cell at around 32-celled embryo stage. The transcripts of *WUS* was detected as early as in 16-celled embryo stage and confined to the four inner cells of the apical region (Mayer et al., 1998; Haecker et al., 2004). However, whether *WOXs* in other plants display a conserved expression pattern in early embryogenesis is still largely unknown. In the present work, cDNA from embryos at successive stages (from stage 1 to stage 9) were prepared for RT-qPCR according to our previous protocol (Zhao et al., 2013). Generally, 10 *WOXs* could be detected in embryos at one or more stages. Similar to *WOXs* in *A. thaliana, WOXs* in tobacco display a stage-specific expression pattern during the process of embryogenesis (Figure 3). The transcripts of five *WOXs* (*WOX2, WOX9, WOX11, WOX13a*, and *WOX13b*) could be detected in embryos as early as stage 1 (2-celled proembryo stage) and presented in the whole process of embryo development (Figure 3). *WUS* and *WOX5* initiated their transcription in stage 3 (8-celled embryo stage) and stage 4 (32-celled embryo stage), respectively. *WOX 1* initiated its transcription in the middle stages of embryo development (stage 5). *WOX3c* and *WOX3d* initiated their transcription much later than other *WOX* family genes. Their transcripts could only be detected in stage 8 (heart-shaped embryo stage) and stage 9 (torpedo-shaped embryo stage), respectively (Figure 3).

### Parental-of-origin transcripts and *de novo* transcripts of *WOXs* are confirmed in zygote

**Figure 4:**
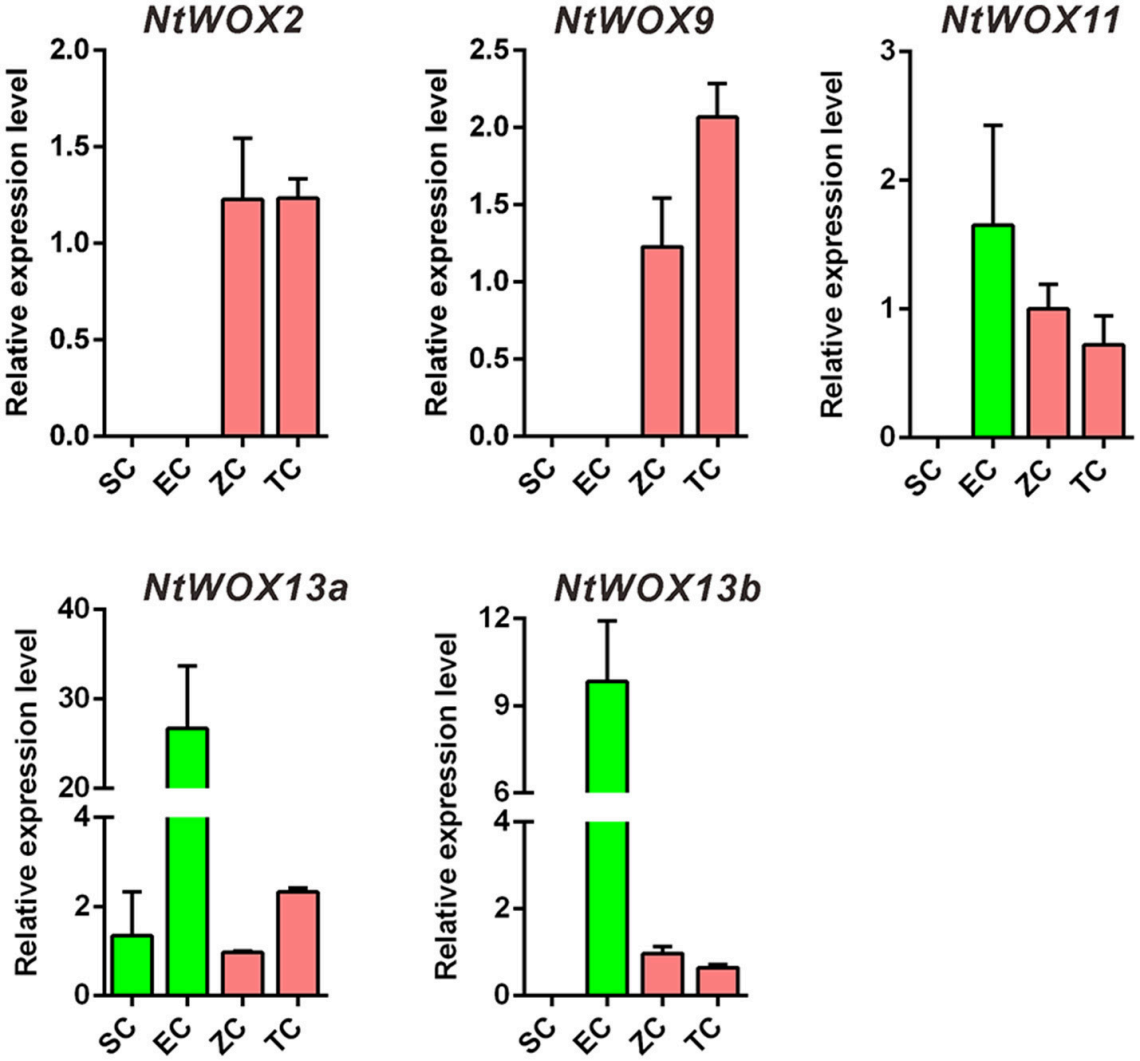
Test of *de novo* or gamete-delivered transcripts in zygote. Relative expression levels of *WOXs* in sperm cells (SC), egg cells (EC), zygote (ZC), and 2-celled proembryos (TC). The expression level of each *WOX* in zygote was set as 1. The expression level was normalized to the average expression level of GAPDH (AJ133422), polyubiquitin (GQ281244), and elongation factor 1α (AF120093). Error bars represent ± standard error from three independent experiments.

Unlike animals, in which zygote genome is quiescent and maternally derived factors deposited in egg cell are enough to drive early embryo development, plant zygotes are transcriptionally active and begin to transcribe to generate transcripts for early embryo development (Ning et al., 2006; Zhao et al., 2011, 2017; Del Toro-De Leon et al., 2014). Early expression pattern analysis of *WOXs* in *A. thaliana* revealed that *WOX2* and *WOX8* were co-expressed in the zygote, and *WOX9* initiated its transcription in the basal cell (Haecker et al., 2004). Recently, both paternal factors SSP and maternal factors HDG11/12 were reported to regulate the expression of *WOX8* in the zygote, which is critical for embryo pattern formation (Ueda et al., 2017). Whether *WOX* transcripts in zygote is delivered by gametes or *de novo* transcribed is not clear. To answer this question and test whether the expression of *WOXs* in gametes and early embryos are similar to that in *A. thaliana*, the relative expression level of *WOXs* in sperm cells, egg cells, zygotes, and 2-celled proembryos were quantified (Figure 4). Unlike *A. thaliana*, the transcripts of *WOX2* was firstly detected in zygotes, but not in egg cells and sperm cells (Figure 4). *WOX9* displays a similar expression pattern as *WOX2*, which initiated its transcription as early as in zygote, indicating their *de novo* transcription rightly after fertilization. More interestingly, both *WOX11* and *WOX13b* show high expression levels in egg cells, but decrease significantly in zygotes. This specific expression pattern of *WOX11* and *WOX13b* suggests that their transcripts in zygote are likely delivered by egg cells.

### Uneven expression of *WOXs* in apical and basal cell lineages

**Figure 5:**
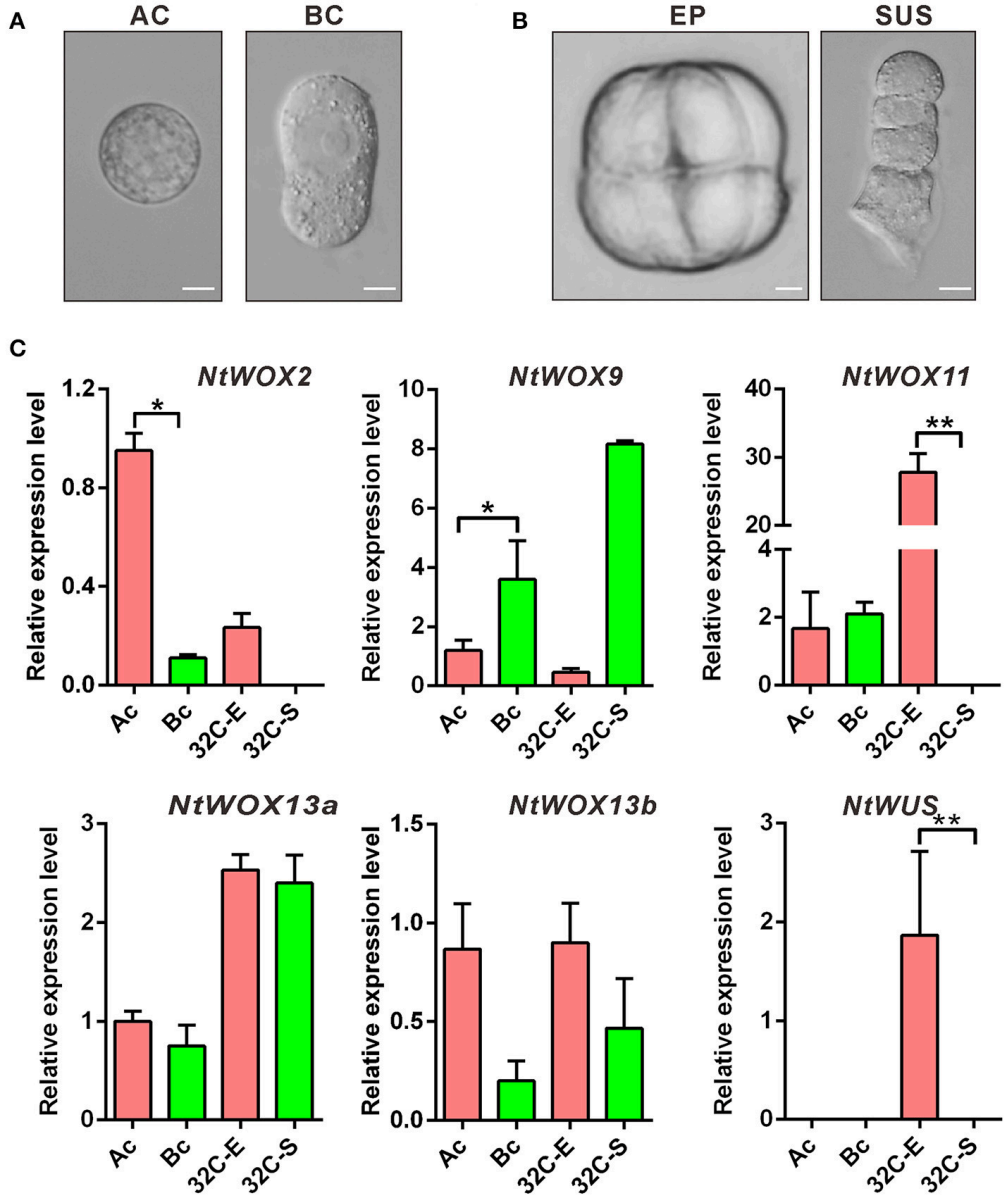
Detection of cell type-specific expression of *WOXs*. **(A)** Isolation of apical cell (AC) and basal cell (BC), respectively. Bar = 10 μm. **(B)** Isolation of embryo proper (EP) and four-celled suspensor (SUS) at 32-celled embryo stage, respectively. Bar = 10 μm. **(C)** Relative expression level of each *WOX* in apical cell linages and basal cell linages. The expression level of each *WOX* (Except *WUS*) in the apical cell was set as 1. The expression level was normalized to the average expression level of GAPDH (AJ133422), polyubiquitin (GQ281244), and elongation factor 1α (AF120093). Error bars represent ± standard error from three independent experiments. Ac, apical cell; Bc, basal cell; 32C-E, embryo proper at 32-celled embryo stage; 32C-S, suspensor at 32-celled embryo stage.

First asymmetric zygote division is one of the critical cell divisions in early embryogenesis, which usually gives rise to the formation of two daughter cells with different division pattern and distinct developmental fates. Previous transcription profile analysis revealed that asymmetric zygote division resulted in uneven distribution of some specific transcripts in apical and basal cell, respectively (Ma et al., 2011). Similar transcription profile analysis of apical and basal cell has not been performed in *Arabidopsis*, possibly due to the difficulty in isolating small living apical cell and basal cell. However, expression pattern analyzed by *in situ* hybridization revealed that different *WOX*s display cell type-specific expression pattern. *WOX2* and *WOX8* were found to be confined to the apical cell and basal cell after zygote division, respectively. *WOX9* was firstly detected in the basal cell and subsequently shifted to the descendants of the apical cell (Haecker et al., 2004). To test whether *WOXs* also show a similar pattern in apical and basal cell lineages of early embryo in tobacco, apical cell and basal cell linages of 2-celled proembryo (Figure 5A) and around 32-celled embryo (Figure 5B) were isolated for mRNA extraction and cDNA synthesis, respectively. RT-qPCR was used to compare their relative expression levels in apical and basal cell linages. Surprisingly, none of them are exclusively expressed in apical cell or basal cell. The transcripts of *WOX2* and *WOX9* could be detected in both apical and basal cell. However, *WOX2* show a significant higher expression level in apical cell compared with that in basal cell (>8-fold), and become confined to embryo proper at 32-celled embryo stage. *WOX9* show a significant higher expression level in basal cell (>2-fold compared with that in apical cell) and suspensor cells (>20-fold compared with that in embryo proper), indicating that the transcription of *WOX2* and *WOX9* was gradually restricted to the apical or basal cell linages as the embryo develop, respectively. More interestingly, the expression of *WOX11* was also confined to embryo proper at 32-celled embryo stage, and *WUS* initiated its transcription specifically in embryo proper (Figure 5). Thus, despite no specific expression of *WOX* family genes in apical or basal cell, the expression pattern of *WOXs* in apical and basal cell linages of around 32-celled embryo implies a relatively conserved mechanism for apical-basal embryo polarity establishment in both *N. tabacum* and *A. thaliana*.

### WUS-box is an evolutionarily conserved motif for the repressive activity of WOXs

**Figure 6:**
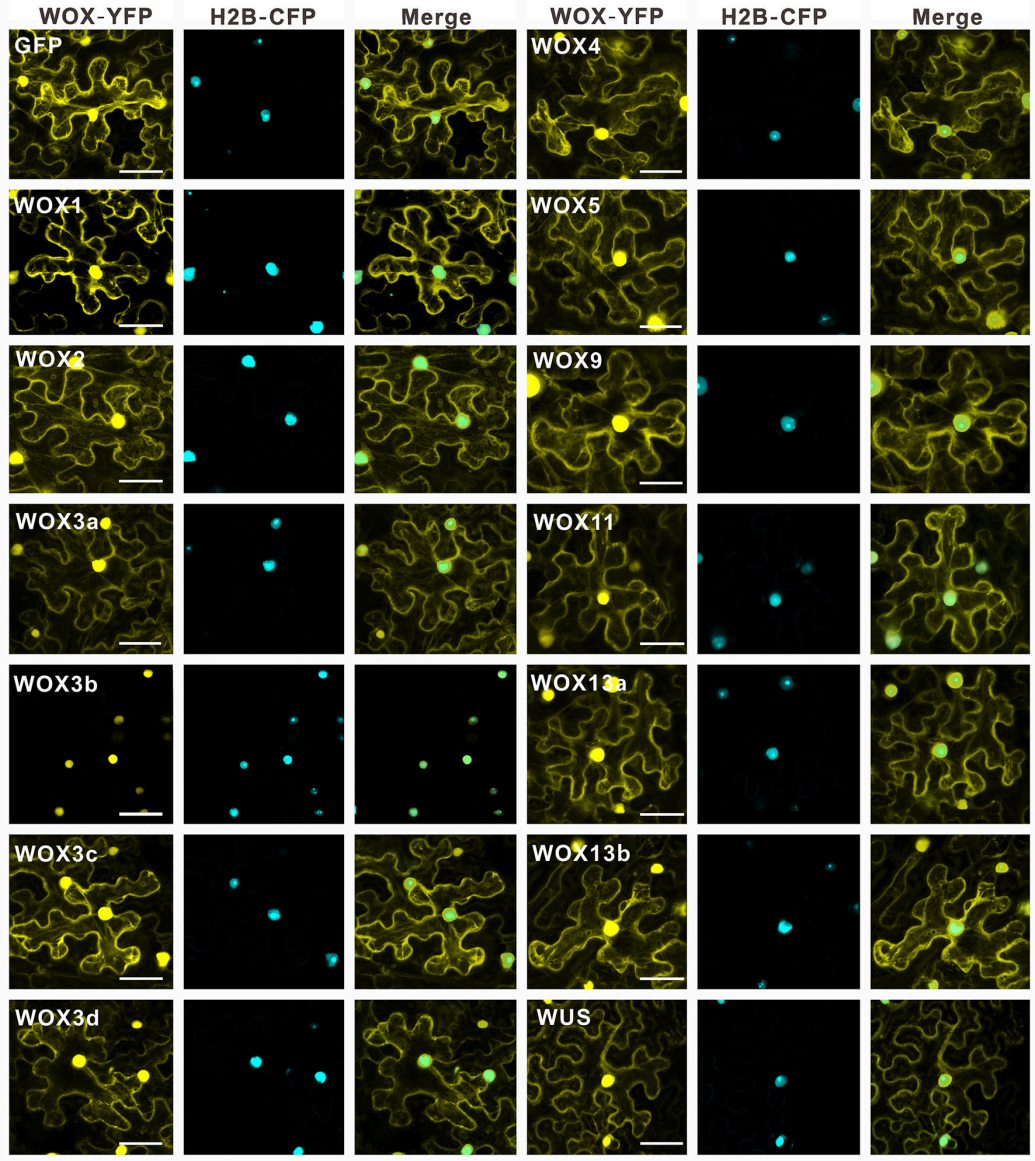
Subcellular location analysis of WOX family proteins in *N. benthamiana* leaves. YFP alone was used as a control. Bar = 50 μm.

Early reports in *A. thaliana* revealed that WUS had the strong repressive activity to suppress the expression of the reporter gene due to its WUS box (Ikeda et al., 2009). Recently, research about *Medicago truncatula WOX* gene, *STENOFOLIA (STF)*, further confirmed that transcriptional repressor activity of STF was conferred by WUS box in the C-terminal (Lin et al., 2013). However, the transcriptional activities of all WOX member in a certain species have not been determined. And whether the role of WUS box for transcriptional repressor activity is conserved among different plants is still largely unknown. To determine which WOXs in tobacco acts as a transcriptional activator or repressor, the intracellular localization of each WOX was firstly investigated. The construct *35S::WOX-YFP* for each *WOX* was generated respectively. And the construct *35S::YFP* was chosen as a control. The results revealed that all WOXs could be detected in the nucleus, corresponding to the characteristics of transcriptional factors (Figure 6).

**Figure 7:**
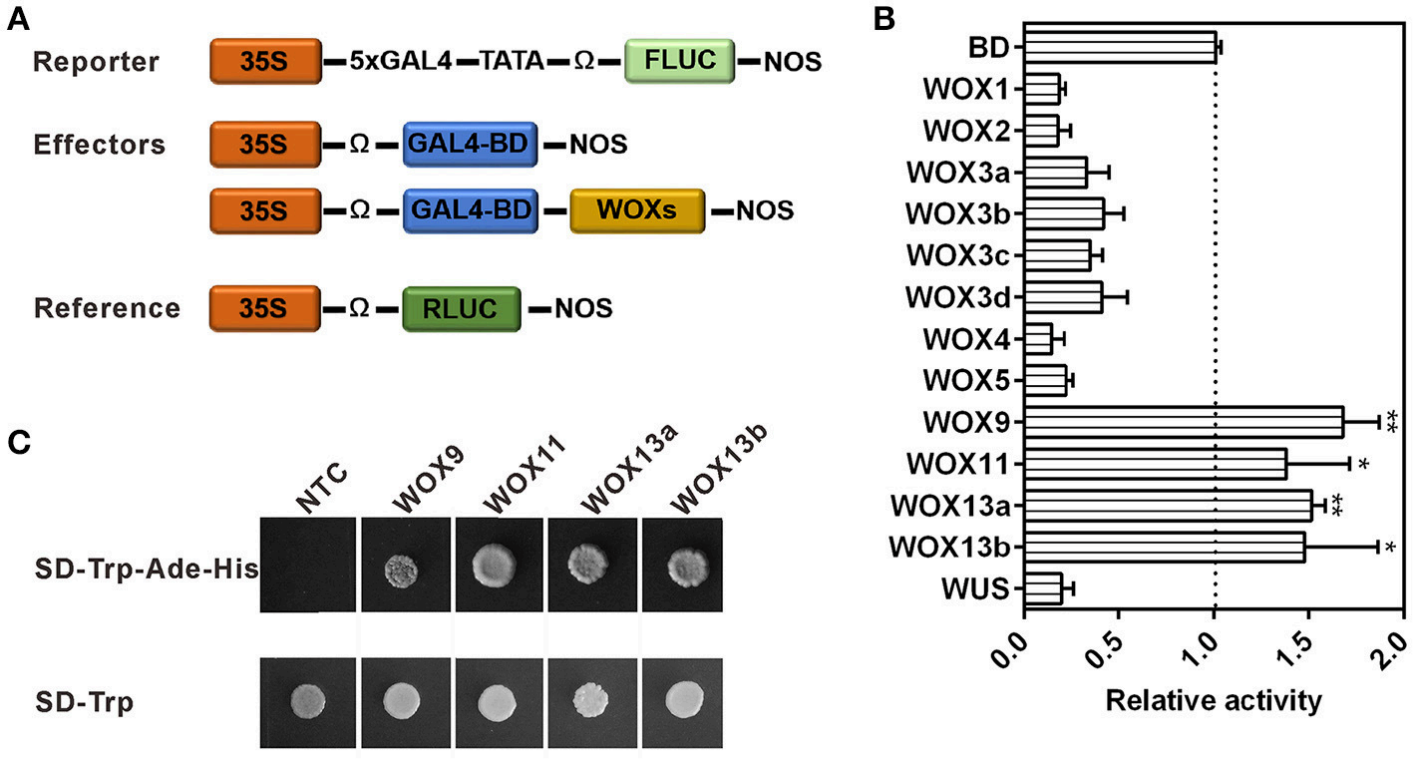
The repressive or activation activities of WOXs in tobacco. **(A)** Reporter, effector, and reference constructs used in transient expression analysis. **(B)** Relative luciferase activities of each effector compared with GAL4-DB control. Error bars indicate standard error from four independent experiments. ^*^ and ^**^ indicate statistical difference compared to the control (*t*-test, *p* < 0.05, or *p* < 0.01, respectively). **(C)** The transcriptional activation activities of WOXs in yeast.

Next, we examined their transcriptional activities through luciferase transient expression assays in leaf epidermis of *N. benthamiana*. The effector plasmid contained each *WOX* fused with GAL4 DNA-binding domain, and the reporter plasmid contained a 5×GAL4-binding site fused with the luciferase (LUC) gene (Figure 7A). Bioluminescence measurements revealed all WOXs in WUS clade could reduce luciferase activities (>2-fold), indicating strong repressive activity of these WOXs. However, four WOXs (WOX9 and WOX11 in the intermediate clade, WOX13a and WOX13b in ancient clade) led to the significant increase of luciferase activities, indicating strong activation activities of these four WOXs (Figure 7B). By comparison of these WOX protein sequences, we found these four WOX proteins lack typical WUS-box motif “TLXLFPXX,” suggesting that WUS-box in WOX proteins are likely responsible for their repressive activities. More interestingly, WOX11 in intermediate clade with a WUS box-like motif “TNXLFPXX” (with a substitution in the second position of the motif) in the upstream of typical WUS box also displayed no ability to repress the luciferase activities (Figure S5), suggesting that the core amino acids of WUS box or the position of WUS box-like motif in WOX protein is critical for the repressive activity of WOXs (Figure S5). To confirm this result, four WOXs with activation abilities were fused to the GAL4 DNA-binding domain in pGBKT7 and transformed into yeast strain to test their abilities for transcriptional activation. Consistent with the result of luciferase transient expression assay, these four WOXs can activate the expression of reporter genes in yeast (Figure 7C). These results suggest that the original WOXs have transcriptional activation capacity, whereas repressive ability was equipped later through the acquisition of WUS-box as a repressive motif in the C-terminal of the protein. Consistent with this hypothesis, no WUS-box motif could be detected in ancient WOXs from unicellular green algae *Ostreococcus lucimarinus* and *Ostreococcus tauri*, moss *Physcomitrella patens* and spikemoss *Selaginella moellendorffii* (Figure S8). However, which motif contribute to the transcriptional activation ability of WOXs in ancient and intermediate clade is still unknown.

## Discussion

### Main characteristics of *WOX* family genes in tobacco

WUSCHEL-related homeobox genes, a plant-specific clade of homeobox transcription factors, have been identified in plants from different clans (Mukherjee et al., 2009). Researches revealed that *WOXs* in *A. thaliana* have important roles in different developmental processes, especially in embryo development including cell fate determination of apical and basal cell lineages and the establishment of the apical-basal pattern (Haecker et al., 2004; Breuninger et al., 2008). However, variations in their expression pattern and roles among different plant species are still largely unknown. *N. tabacum* has long been considered as an ideal model plant for embryogenesis, which displays a highly stereotyped and predictable cell division pattern, beginning from the first asymmetric zygote division to embryo maturation. Furthermore, unlike in *A. thaliana*, relevant techniques for the isolation of gametes and early embryos in tobacco have been well-established, which greatly facilitate the investigations of detailed expression pattern of *WOXs* (He et al., 2007; Zhao et al., 2013, 2016). In this work, 13 *WOXs* have been identified and could be divided into three groups based on phylogenetic analysis. Phylogenetic analysis revealed significant differences not only in *WOXs* between eudicots and monocots clade, but also in eudicots clade. First divergence is that no *AtWOX8* orthologs was found in monocots and eudicots except *A. thaliana*. In addition, there is no evidence that monocots clade contain orthologs of *WOX 1, 6, 7, 10*, and *14*. Surprisingly, the majority orthologs (except *WOX1*) that disappeared in monocots clade are also lost in other eudicots, suggesting that *A. thaliana* genome generate some species-specific *WOXs* during the course of the evolution. Another significant divergence is that two tobacco *WOXs* (*NtWOX3* and *NtWOX13*) have multiple paralogs derived from chromosomal duplication. Duplicated *WOXs* have also been found in other plants such as *WOX2* and *WOX5* in maize (Figure S3 and Table S1). Comparable analysis of these WOXs indicated the composition of protein domain and transcriptional activities within each class are similar, suggesting possible functional redundancy of *WOXs* in same group (Figure S4 and Figure 7).

### Structure base of WOXs for regulating the transcription of their targets

The function of WOXs in relation to their specific structure has been discussed in previous works. Among them, *WUS*, the founding member of WOX family, was approved to mainly act as a repressor of transcription in the maintenance of stem cell identity, but also act as a direct activator of *AG* gene in flowers (Lohmann et al., 2001). The transcriptional repressive activity of WUS was conferred by the WUS-box in the C-terminal. Similar transcriptional repressor activity has also been found in *M. truncatula WOX* gene, *STF* (Lin et al., 2013). However, the relationship between transcriptional activities of WOXs and their protein characteristics has not been comprehensively analyzed. Here, nine of 13 tobacco WOXs have obvious transcriptional repressive activities, whereas other four WOXs display strong activation activities. By comparison of nine WOX protein sequences, a typical WUS-box motif “TLXLFPXX” was identified in the C-terminal of them. Whereas, other four WOXs with transcriptional activation activities lack typical WUS-box motif, suggesting that WUS-box in tobacco WOXs are likely responsible for their repressive activities. More interestingly, phylogenetic analysis results revealed that all nine WOXs with transcriptional repressive activities fall into WUS clade, whereas WOXs with transcriptional activation activities fall into ancient clade or intermediate clade, suggesting that the original WOXs likely act as transcriptional activation factors, whereas transcriptional repressive ability was equipped later through the acquisition of functional WUS-box motif in the process of WOX evolution.

HD in WOXs is an evolutionarily conserved DNA-binding domain, which is composed of ~60 amino acids that fold into a flexible N-terminal arm and a stable three-helix bundle. Researches about HOX genes in animals revealed that the DNA binding site of HD is usually formed by the N-terminal arm in the minor groove and a single “recognition” helix in the major groove (Noyes et al., 2008). Despite a common DNA-binding structure, amino acid variations in HD have also been found. Residues both in N-terminal arms and recognition helix contributed to their DNA-binding specificity. Relationship between binding specificities and amino acid sequence in DNA-binding architecture of 84 independent homeodomains from *D. melanogaster* have been comprehensively analyzed, allowing the prediction of preferred recognition sites of different HOXs from other species (Noyes et al., 2008). However, whether HDs in WOXs display a similar DNA-binding specificity attributed by amino acid sequence in DNA-binding architecture are largely unknown. Homology modeling of HDs revealed that HD in tobacco WOXs display a relatively conserved structure (Figure S7). Alignment of these HDs with that from *D. melanogaster* revealed a relatively conserved amino residues on N-terminal arm and recognition helix, suggesting a potential conserved mechanism of HDs in WOXs recognizing their targeted DNA sequences. Although similar researches on the analysis of HD specificities have not been performed in plants, the “TTAATGG” motif has been recognized by different WOXs from different plants. In *A. thaliana*, WUS could bind to the “TTAATGG” motif in *AG* and *RR* genes (Lohmann et al., 2001; Leibfried et al., 2005). In rice, WOX family proteins QHB, WOX3, and WOX11 have also been shown to be able to interact with the same DNA sequence in different targets (Kamiya et al., 2003; Dai et al., 2007; Zhao et al., 2009). Taken together, “TTAATGG” seems to be a consensus binding motif for different WOXs. However, whether different WOX members could recognize other DNA motifs and display recognition specificities as shown in different HDs from *D. melanogaster* are largely unknown. Hence, comparison of recognition specificities conferred by HDs in different WOXs might be a charming work for understanding molecular mechanism of WOXs in the further study.

### Commonalities and differences in the expression pattern of *WOXs* during early embryogenesis

In *A. thaliana, WOX* family genes are well-known for their specific expression pattern in early embryogenesis. *WOX2* became confined to the apical cell linages, whereas *WOX8* was restricted in the basal cell linages after zygote division (Haecker et al., 2004). *WUS* and *WOX5* were found to be exclusively located in SAM and RAM after around 32-celled embryo stage, respectively. Similar expression pattern of *WUS* and *WOX5* in SAM and RAM was also found in maize (Nardmann and Werr, 2006; Nardmann et al., 2007). Consistent with this observation, *WOX5* initiated its transcription in suspensor at 8-celled embryo stage, and *WUS* was firstly detected in embryo proper at 8-celled embryo stage, suggesting a relatively conserved molecular mechanism for SAM and RAM establishment in both monocots and eudicots. However, some variations in the expression pattern of *WOXs* between monocots and eudicots have also been found, especially about *WOX2* and *WOX8* at 2-celled proembryo stage. The first striking difference is that no *WOX8* has been identified in the genomes of monocots and other eudicots to date. The second is that delayed *WOX2* expression was found in monocots (Nardmann and Werr, 2006; Nardmann et al., 2007; Zhao et al., 2017). Consistent with preceding observations, no homolog of *WOX8* has been identified in tobacco genome. On the other hand, the transcripts of *WOX2* could be detected in both the apical cell and basal cell simultaneously, unlike that in *A. thaliana*. However, the expression of *WOX2* in apical cell is significantly higher than that in the basal cell (>8-fold), and became restricted in the embryo proper at the 32-celled embryo stage. All these results implied that the expression pattern of *WOX* family genes has undergone modifications in the course of evolution, not only in the divergence of eudicots and monocots, but also in different eudicots.

According to the works in *A. thaliana, WOXs* such as *WOX 2, WOX 8*, and *WOX 9* play essential roles in cell fate specification and early embryo patterning. Then, an interesting question raises, are these mechanisms initiated and directed by parental instructions or *de novo* established after fertilization? To answer the question it is necessary to understand the expression behavior of these *WOXs* before and after fertilization. In the present work, the transcripts of *WOX2* and *WOX 9* was only detected in the zygotes, but not in egg cells or sperm cells, suggesting that they are *de novo* transcribed in the zygote, and the relevant mechanism is fertilization-initiated. Interestingly, *WOX11* and *WOX13b* were detected only in the egg cell, but not in the sperm cell, indicating at least in early zygote they show parent-of-origin characteristics. How these maternal transcripts act in early zygote development or initiation of embryogenesis will deserve extensive investigations. Taken together, these *WOXs* may function successively during the transition from gametophytic to the sporophytic generation and link the developmental signaling from parents to offspring.

## Accession numbers

Sequence data for WOX family genes in tobacco has been deposited in GenBank (http://www.ncbi.nlm.nih.gov/Genbank) under the following accession numbers: NtWOX1 (MG843879), NtWOX2 (MG843880), NtWOX3a (MG843881), NtWOX3b (MG843882), NtWOX3c (MG843883), NtWOX3d (MG843884), NtWOX4 (MG843885), NtWOX5 (MG843886), NtWOX9 (MG843887), NtWOX11 (MG843888), NtWOX13a (MG843889), NtWOX13b (MG843890), NtWUS (MG843891).

## Author contributions

Conceived and designed the experiments: PZ and MS. Performed the experiments: XZ, YG, and PZ. Analyzed the data: XZ, YG, and PZ. Wrote the paper: PZ and MS.

### Conflict of interest statement

The authors declare that the research was conducted in the absence of any commercial or financial relationships that could be construed as a potential conflict of interest. The reviewer XZ and handling Editor declared their shared affiliation.
