# Supplementary figures and images for: Comparative Analysis of WUSCHEL-Related Homeobox Genes Revealed Their Parent-of-Origin and Cell Type-Specific Expression Pattern During Early Embryogenesis in Tobacco
Source: Front Plant Sci. 2018 Mar 8;9:311. doi: 10.3389/fpls.2018.00311 (PMC5890105; doi:10.3389/fpls.2018.00311)

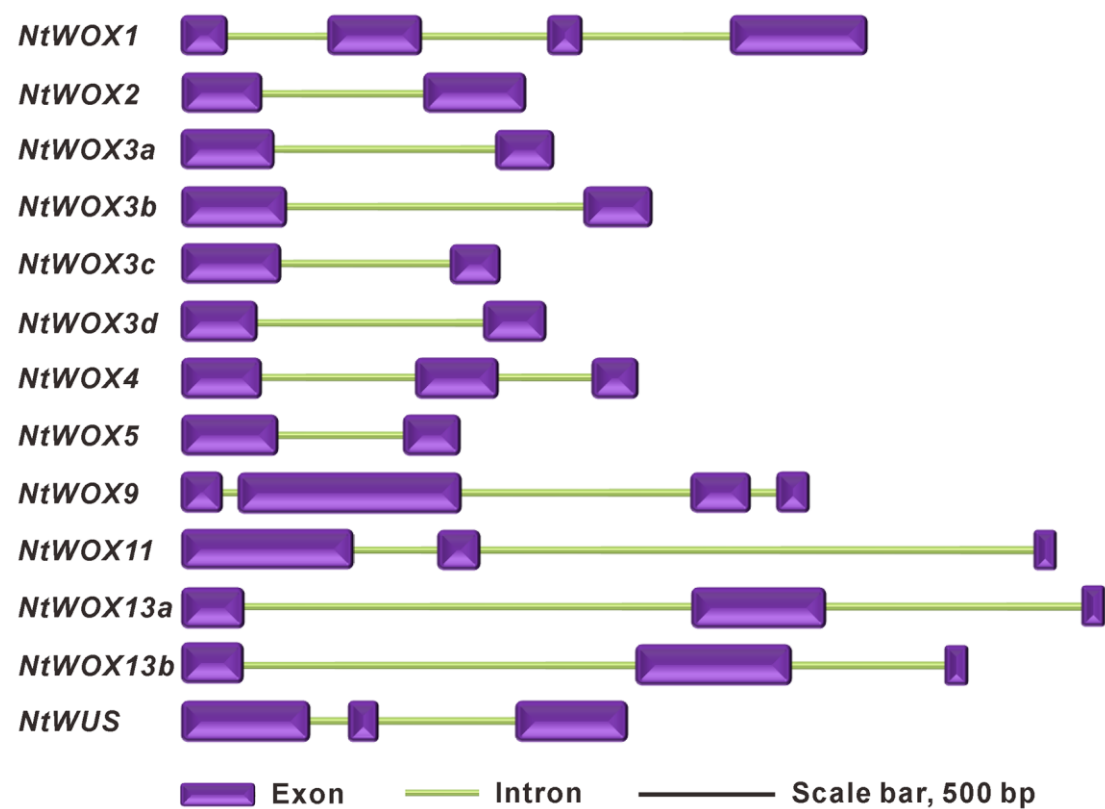

**Figure S1. Genomic structures of WOX family genes in tobacco**

Supplement: Supplementary file 1 [file Image1.PDF]
