# Supplementary material for: Comparative Analysis of WUSCHEL-Related Homeobox Genes Revealed Their Parent-of-Origin and Cell Type-Specific Expression Pattern During Early Embryogenesis in Tobacco
Source: Front Plant Sci. 2018 Mar 8;9:311. doi: 10.3389/fpls.2018.00311 (PMC5890105; doi:10.3389/fpls.2018.00311)
Supplement: Supplementary file 4 [file Image4.PDF]

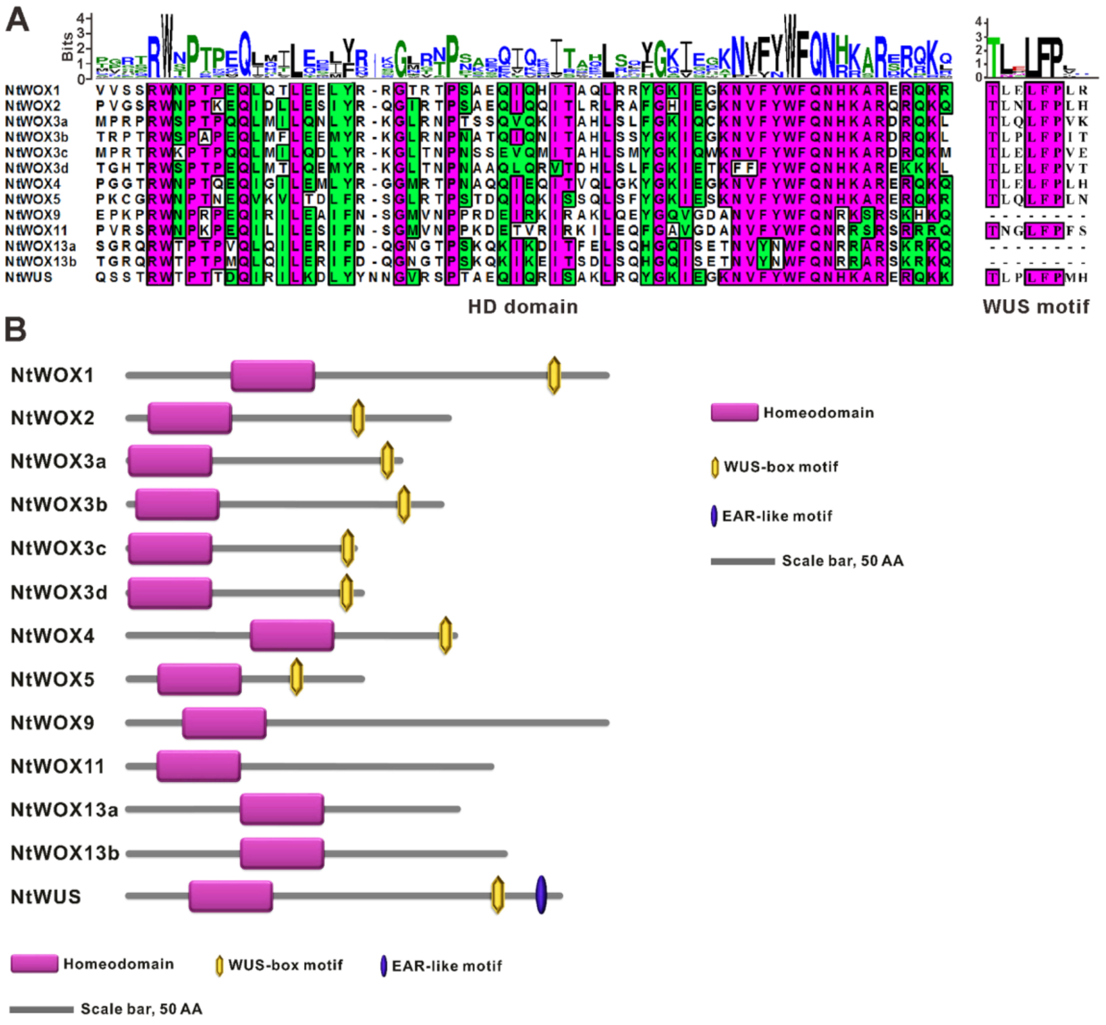

Figure S4. Conserved domain in WOX family protein sequences

A. Protein sequence alignment of WOX protein sequences in tobacco. Identical residues are outlined and shaded orchid. Similar residues are outlined and shaded green. **B.** Functional motifs of WOX family proteins
