# Supplementary material for: Comparative Analysis of WUSCHEL-Related Homeobox Genes Revealed Their Parent-of-Origin and Cell Type-Specific Expression Pattern During Early Embryogenesis in Tobacco
Source: Front Plant Sci. 2018 Mar 8;9:311. doi: 10.3389/fpls.2018.00311 (PMC5890105; doi:10.3389/fpls.2018.00311)
Supplement: Supplementary file 5 [file Image5.PDF]

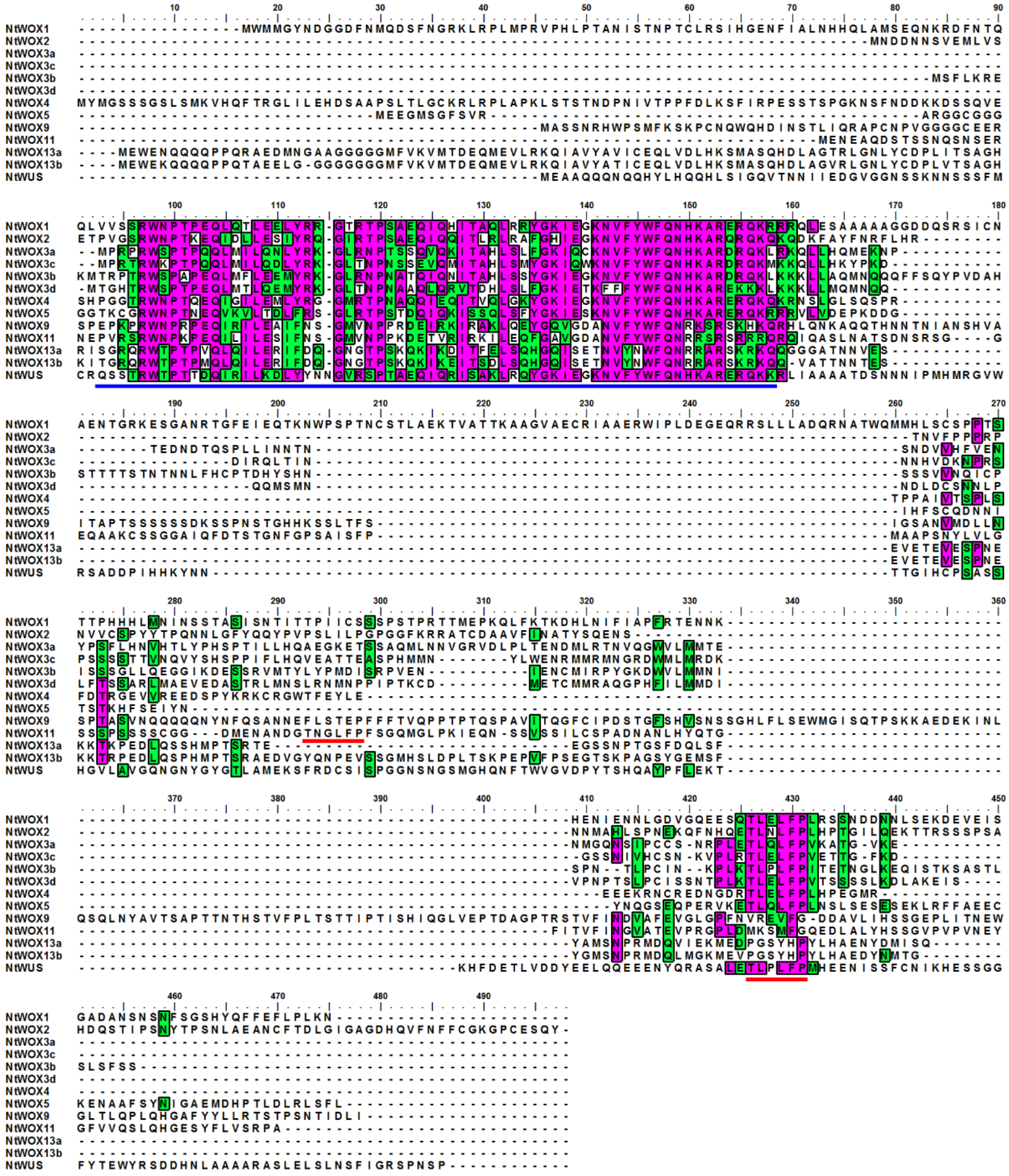

Figure S5. Sequence alignment of WOX protein sequences in tobacco

Identical residues are outlined and shaded orchid. Similar residues are outlined and shaded green. HD domain was labelled with blue line, and WUS-like motif in WOX11 was labelled with red line.
