# Supplementary material for: Comparative Analysis of WUSCHEL-Related Homeobox Genes Revealed Their Parent-of-Origin and Cell Type-Specific Expression Pattern During Early Embryogenesis in Tobacco
Source: Front Plant Sci. 2018 Mar 8;9:311. doi: 10.3389/fpls.2018.00311 (PMC5890105; doi:10.3389/fpls.2018.00311)
Supplement: Supplementary file 6 [file Image6.PDF]

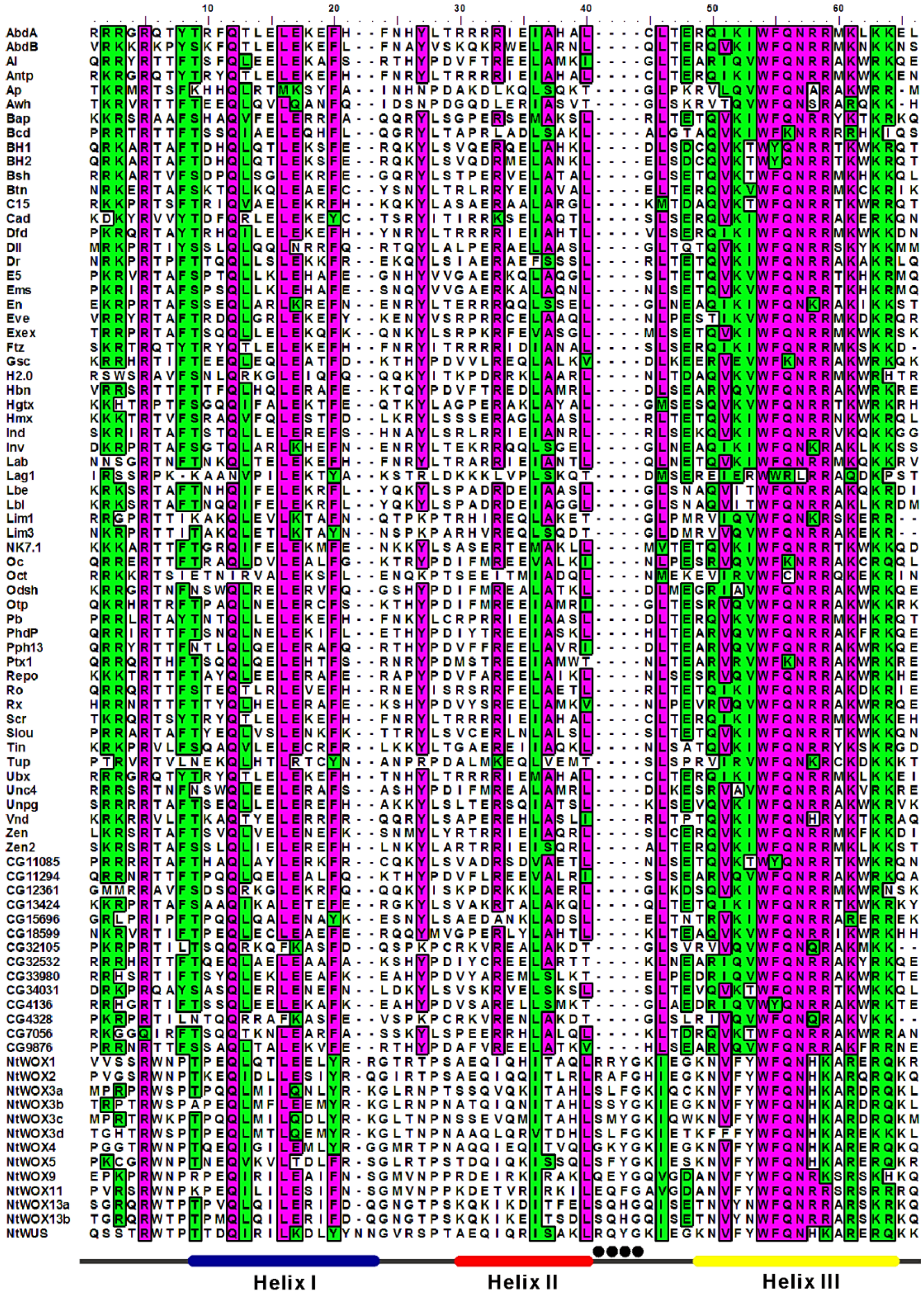

Figure S6. Sequence alignment of homeodomains from the *D. melanogaster* and *N. tabacum*

Identical residues are outlined and shaded orchid. Similar residues are outlined and shaded green. Four-amino-acid loop extension in WOXs from tobacco was labelled black dot.
