# Supplementary material for: Comparative Analysis of WUSCHEL-Related Homeobox Genes Revealed Their Parent-of-Origin and Cell Type-Specific Expression Pattern During Early Embryogenesis in Tobacco
Source: Front Plant Sci. 2018 Mar 8;9:311. doi: 10.3389/fpls.2018.00311 (PMC5890105; doi:10.3389/fpls.2018.00311)
Supplement: Supplementary file 7 [file Image7.PDF]

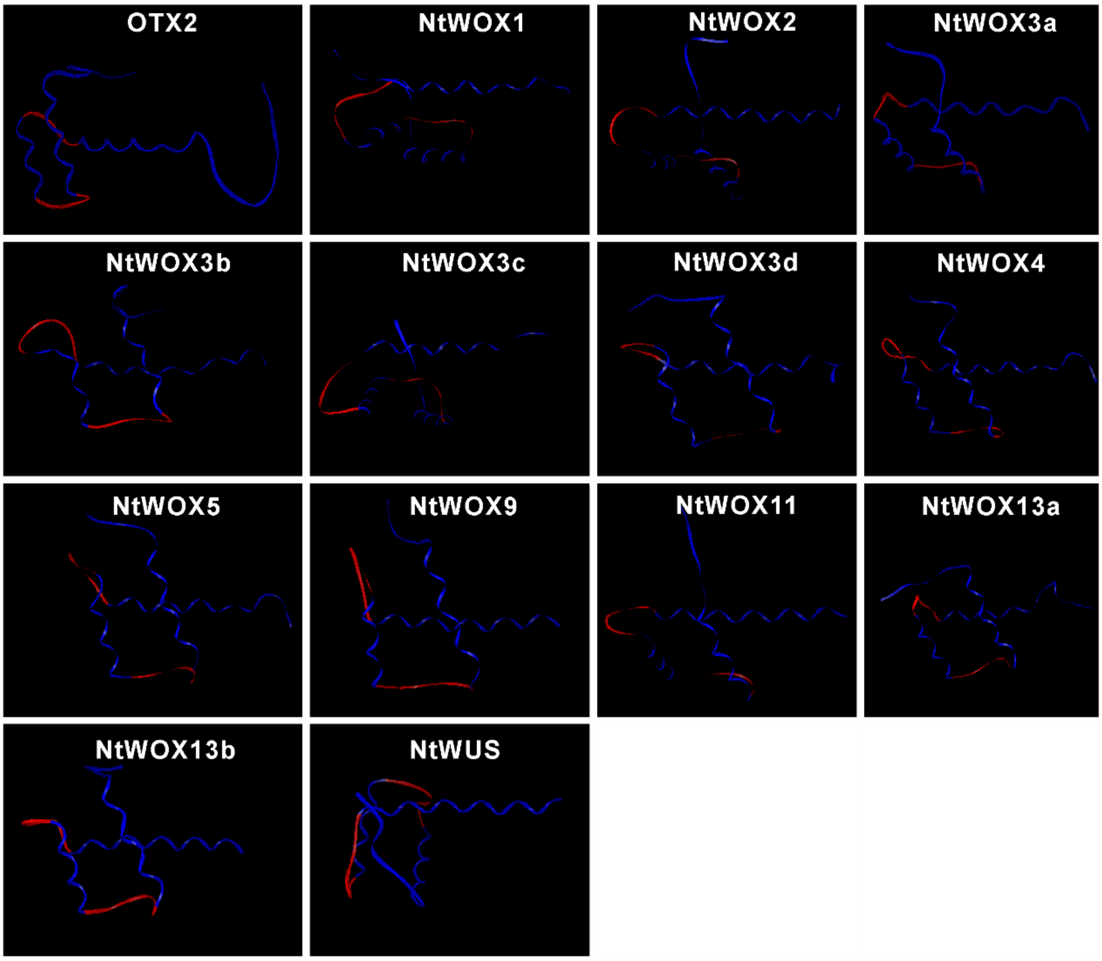

Figure S7. Predication of three-dimensional structures of WOX family proteins in tobacco

Red color indicated additional amino acids detected in HDs.
