# Supplementary material for: Comparative Analysis of WUSCHEL-Related Homeobox Genes Revealed Their Parent-of-Origin and Cell Type-Specific Expression Pattern During Early Embryogenesis in Tobacco
Source: Front Plant Sci. 2018 Mar 8;9:311. doi: 10.3389/fpls.2018.00311 (PMC5890105; doi:10.3389/fpls.2018.00311)
Supplement: Supplementary file 8 [file Image8.PDF]

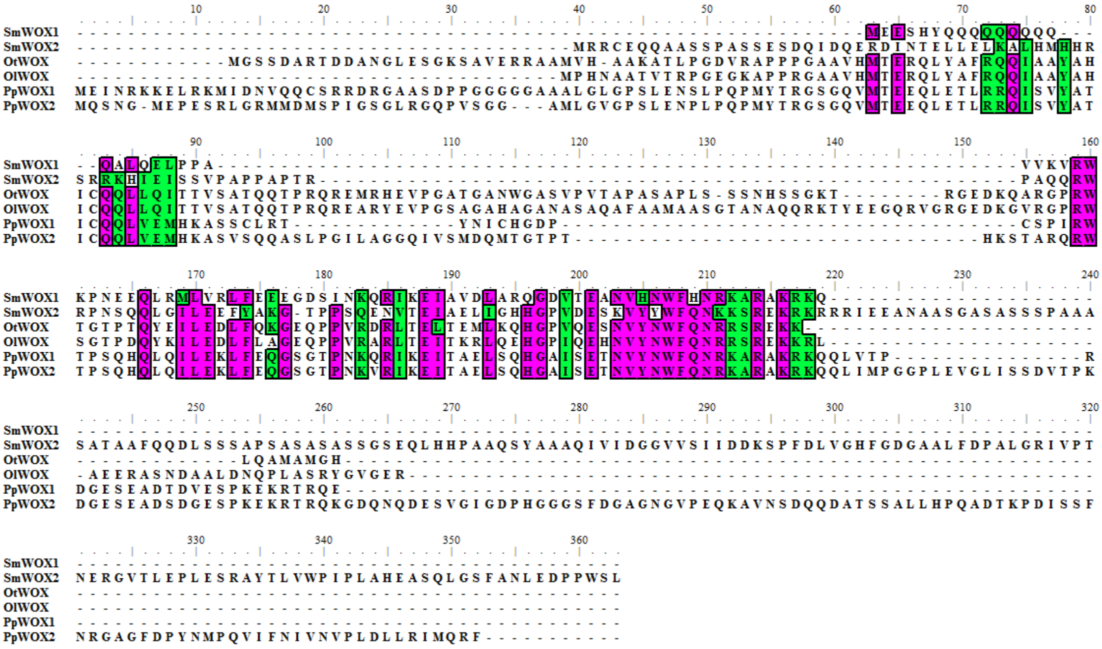

Figure S8. Sequence alignment of homeodomains from green algae *Ostreococcus lucimarinus* (Ol) and *Ostreococcus tauri* (Ot), moss *Physcomitrella patens* (Pp) and spikemoss *Selaginella moellendorffii* (Sm).

Identical residues are outlined and shaded orchid. Similar residues are outlined and shaded green.
