# Supplementary material for: Comparative Analysis of WUSCHEL-Related Homeobox Genes Revealed Their Parent-of-Origin and Cell Type-Specific Expression Pattern During Early Embryogenesis in Tobacco
Source: Front Plant Sci. 2018 Mar 8;9:311. doi: 10.3389/fpls.2018.00311 (PMC5890105; doi:10.3389/fpls.2018.00311)
Supplement: Supplementary file 9 [file Table1.PDF]

Table S1. Sequence information

Species	Name	Accession No.	Species	Name	Accession No.	Species	Name	Accession No.
N. tabacum (13)	NtWOX1		**A.** thaliana (15)	AtWOX1	At3g18010	G. max (17)	GmWOX1a	XM_006583391.1
	NtWOX2			AtWOX2	At5g59340		GmWOX1b	XM_003530910.2
	NtWOX3a			AtWOX3	At2g28610		GmWOX2a	XM_003539041.2
	NtWOX3b			AtWOX4	At1g46480		GmWOX2b	XM_003541976.2
	NtWOX3c			AtWOX5	At3g11260		GmWOX2c	XM_003547327.1
	NtWOX3d			AtWOX6	At2g01500		GmWOX3	XM_006602517.1
	NtWOX4			AtWOX7	At5g05770		GmWOX4a	NM_001254491.2
	NtWOX5			AtWOX8	At5g45980		GmWOX4b	XM_006601216.1
	NtWOX9			AtWOX9	At2g33880		GmWOX5a	XM_006601892.1
	NtWOX11			AtWOX10	At1g20710		GmWOX5b	XM_003518358.2
	NtWOX13a			AtWOX11	At3g03660		GmWOX9a	XM_003541466.2
	NtWOX13b			AtWOX12	At5g17810		GmWOX9b	NM_001255876.1
	NtWUS			AtWOX13	At4g35550		GmWOX11	XM_003520903.2
				AtWOX14	At1g20700		GmWOX13a	XM_003536718.2
				AtWUS	At2g17950		GmWOX13b	XM_003523000.2
							GmWOX13c	XM_006574301.1
							GmWUS	XM_003517132.2

O. sativa (11)	OsWOX2	Os01g0840300	S. bicolor (11)	SbWOX2	XM_002458694.1	Z. mays (17)	ZmWOX2a (ZmWOX5)	XM_008658856.1
	OsWOX3	AB218893.1		SbWOX3a	XM_002448974.1		ZmWOX2b (ZmWOX5)	XM_008676620.1
	OsWOX4	Q7XTV3.2		SbWOX3b (SbWOX2)	XM_002440454.1		ZmWOX3a	AJ536578.1
	OsWOX5 (OsWOX9)	Os01g0854500		SbWOX4	XM_002448595.1		ZmWOX3b	AM491777.1
	OsWOX9a (OsWOX7)	Q0JKK6.2		SbWOX5 (SbWOX9)	XM_002458736.1		ZmWOX3c	AM490236.1
	OsWOX9b (OsWOX12)	Os05g0564500		SbWOX9 (SbWOX12)	XM_002440209.1		ZmWOX4	AM490237.1
	OsWOX11a (OsWOX10)	Os08g0242400		SbWOX11a (SbWOX10)	XM_002444050.1		ZmWOX5a (ZmWOX9)	XM_008676586.1
	OsWOX11b	Os07g0684900		SbWOX11b	XM_002461189.1		ZmWOX5b (ZmWOX9)	XM_008658821.1
	OsWOX11c (OsWOX6)	Os03g0325600		SbWOX11c (SbWOX6)	XM_002465290.1		ZmWOX9a	AM490240.1
	OsWOX13 (OsWOX8)	Os01g0818400		SbWOX13 (SbWOX8)	XM_002456495.1		ZmWOX9b (ZmWOX7)	AM490241.1
	OsWUS	AM234746.1		SbWUS	XM_002448662.1		ZmWOX9c (ZmWOX12)	XM_008651789.1
							ZmWOX11a	XM_008655268.1
							ZmWOX11b	EU954172.1
							ZmWOX11c	NM_001279514.1

					ZmWOX13 (ZmWOX14)	EU961475.1
					ZmWUS1	AM234744.1
					ZmWUS2	AM234745.1
