# Supplementary material for: Comparative Analysis of WUSCHEL-Related Homeobox Genes Revealed Their Parent-of-Origin and Cell Type-Specific Expression Pattern During Early Embryogenesis in Tobacco
Source: Front Plant Sci. 2018 Mar 8;9:311. doi: 10.3389/fpls.2018.00311 (PMC5890105; doi:10.3389/fpls.2018.00311)
Supplement: Supplementary file 10 [file Table2.PDF]

Table S2. Primers used in the present study

WOX	For RT-PCR		For RT-qPCR	
	Forward (5’-3’)	Reverse (5’-3’)	Forward (5’-3’)	Reverse (5’-3’)
NtWOX1	ACAAGGCTAGGGAACGCCAAAAAC	GTGGGAGGTGAACAAGACAAATGC	GCAACAACAAAAGCAGCAGGAGTG	TGCTTTGGCTCCATTGTCGTTC
NtWOX2	GGCAACGCCAGAAACAGAAGCAAG	AACACGAAGCAGAACCCAACCAGA	GCCAGAAACAGAAGCAAGATAAAT	TTGGGCGAGGTGGTGGAAAAAC
NtWOX3a	GAAAAACCCTACTGAAGATAATGATACTC	TACATTACCTCAACGGCCCTACTC	CTTCATAATGTCCATACACTTTACCCT	TCTCTGTCATCATCAAAACCCAAC
NtWOX3b	TACTCTCATAATTCTTCATCTGTTGTGA	AGCTGATTTTGTACTGATCTGTTCC	CAACCTTTTTCACTGCCCTACTG	GATTCATCTTTGATTCCACCCTCT
NtWOX3c	AGATGAAAAAACAGCTTTTGCATAAATACC	TGCAATGGACAATATTAGAAGAACC	GACAAAAACCCTCGTTCTCCTTC	CTACCGTTCATCCTCATCATCCG
NtWOX3d	CATTACAAGAAATGTATAGGAAAGGATTG	CTGGAAGATGTGACAGGGAAGAGC	GGCTAGATTAATGGCAGAAGTTGA	GCTGGAAGATGTGACAGGGAAG
NtWOX4	GCTCCTTCACTTACACTTGGTTG	GATGTCTTTGTTCTTTTCTCTTTCC	GAAGAGGAAAAGAGAAACTGTAGAGAGG	GCAGATGCAAGTAAAAAGATGACG
NtWOX5	GATCAGATACAAAAAATATCTTCCCAAC	AACCTAAAAGCTCAAAAACCATGC	AAGTGAATCGGAGTCAGAGAAGC	CGGAATAAAAAGTACGCCATAAG
NtWOX9	ACAGCAAAGGTAGCAAGCAAGAG	GGAGGATTTATCAGAGGATGAAGAAG	GATGTTGCTTTTGAGGTTGGGTT	CAATAGTGTTGGATGGAGTTGACG
NtWOX11	CCCAAAGATGAGACAGTGAGAATT	AGGGGAAGAAGAACCAAGAACAAG	AACTCTTCTGTCAGCTCAATTTTGT	CTTGGCACCTCTGTCGCTACTC
NtWOX13a	AATTGCAGTGTACGCTGTCATTTG	TGTCAGTATTTTGTCTTGCTTCTTGT	GAAAAGAAAACAAAGCCAGAGGA	TGGATTAGACATTGCATAGAAGGAC
NtWOX13b	TTGAGTCACCCAATGAAAAGAAAAC	CATCAGCGGCTAATGCTAAGATC	GAGGGGGTGGTGGGATGTTTGT	GCGTTGTCTGCCAGTGATTTTAT
NtWUS	TAAGACAGTACGGTAAGATTGAAGGC	GATCTGCCAATGAAAGAGTTGAGAC	CACTATCTTCACCAACAACATTTATCA	AGCCTCTTCTTTTGCCTTTCAC
